# CGM patterns in adults with cystic fibrosis-related diabetes before and after elexacaftor-tezacaftor-ivacaftor therapy

**DOI:** 10.1016/j.jcte.2022.100307

**Published:** 2022-10-01

**Authors:** Hanna Crow, Charles Bengtson, Xiaosong Shi, Leland Graves, Abeer Anabtawi

**Affiliations:** a University of Kansas Medical Center, Department of Internal Medicine, Division of Endocrinology, Metabolism & Clinical Pharmacology, 4000 Cambridge Blvd, Kansas City, KS 66160, USA; b University of Kansas Medical Center, Department of Internal Medicine, Division of Pulmonary, Critical Care and Sleep Medicine, 4000 Cambridge Blvd, Kansas City, KS 66160, USA; c University of Kansas Medical Center, Department of Biostatistics & Data Science, 4000 Cambridge Blvd, Kansas City, KS 66160, USA

**Keywords:** CGM, Cystic fibrosis-related diabetes, CFTR modulator, Elexacaftor-tezacaftorivacaftor (ETI)

## Abstract

Cystic fibrosis-related diabetes (CFRD) is a common complication of cystic fibrosis that is associated with worse outcomes and higher mortality rates. CF transmembrane conductance regulator gene (CFTR) modulators have shown favorable effects on lung function, pulmonary exacerbations, and nutrition status. However, data regarding effects of CFTR modulators on glycemic control among those with CFRD is lacking. In this retrospective study, CGM data was analyzed to determine effect of elexacaftortezacaftor- ivacaftor therapy (ETI), a CFTR modulator, on glucose control among patients with CFRD. No difference was seen in glucose patterns after 3- and 6- months of starting ETI.

## Introduction

Cystic fibrosis (CF) is an autosomal recessive disorder that is caused by abnormalities in the *CF transmembrane conductance regulator* gene (*CFTR*). People with CF have defective copies of the gene resulting in thick, viscous secretions that leads to multi-organ system complications. Cystic fibrosis-related diabetes (CFRD) is one of the most common extrapulmonary comorbidities among people with CF, occurring in up to 50 % of adults. [1] CFRD is a progressive condition that is associated with worse clinical outcomes, decreased pulmonary function, and higher mortality rates. [2] Despite this, the pathogenesis of CFRD remains incompletely understood and is likely multifactorial. Studies show numerous factors associated with progression to CFRD including age, *CFTR* genotype, alterations in the exocrine pancreas, inflammation, loss of β-cell mass and reduced insulin production [3], [4], [5].

A new class of medication, CFTR modulators, are available for select groups of people with CF based upon *CFTR* mutation status. This class of medication differs from traditional symptomatic therapies in that they improve or restore the function of the defective CFTR protein. CFTR modulators have been shown to improve lung function (FEV1), risk of pulmonary exacerbation and nutrition status. [6], [7], [8] Among patients with CFRD, small clinical studies have shown mixed results regarding changes in glycemia with CFTR modulators. [9], [10], [11], [12], [13] Recently, the CFTR modulator elexacaftor-tezacaftor-ivacaftor (ETI) was approved for patients aged 6 years and older who have at least one copy of the most common *CFTR* mutation, *F508del*. This represents 85 % of the cystic fibrosis population. [14] In phase III trials of ETI, the effects on glucose were not analyzed [8], [15].

Continuous glucose monitors (CGM) are small, wearable devices that measure interstitial glucose every 5–15 min to provide glycemic data over 24 h. Although no guidelines exist regarding CGM use in the CFRD population, CGM use is validated in those with CF. [16] CGM has been shown to optimize insulin therapy with favorable effects in BMI and pulmonary function. [17] However, few studies have investigated CGM in CFRD patients on CFTR modulator therapy. [18], [19].

We conducted a retrospective, single-center study in adults 18 years and older with CFRD to determine if ETI has a favorable effect on glycemia by analyzing CGM data. We hypothesized that ETI would improve CGM markers of hyperglycemia.

## Methods

### Participants

Patients 18 years or older with CFRD receiving care at the University of Kansas Medical Center endocrinology clinic, who utilize Dexcom® G6 CGM as part of their routine clinical care and on standard doses of ETI for at least 6 months were included in this retrospective study. Patients were excluded if they did not have minimum 7 days of CGM data in the 3 months prior to starting ETI and within 6 months after starting the drug. Those who were hospitalized or had active infections during the study period were excluded. Patients were identified using the Cystic Fibrosis Foundation Patient Registry (PortCF). All procedures were approved by the University of Kansas Medical Center Institutional Review Board.

### CGM & laboratory measures

Dexcom Clarity® website was used to download glucose data points for each patient for 3 months before and two 3-month periods after starting ETI. Chart review was performed to collect clinical information 3 months before and two 3-month periods after starting ETI including FEV1 % predicted, body mass index (BMI), hemoglobin A1c, and insulin dosing. Due to limitations of chart review, we were not able to collect information regarding duration of diabetes. We were not able to collect accurate short acting insulin dosing on those using multiple daily injections (2 patients), thus only basal insulin dose was reported. In those using insulin pump therapy (9 patients), average total daily insulin dosing was obtained from pump download reports scanned in electronic medical records when available. Only basal insulin dose was obtained from office visit documentation when pump download was not available. One patient on pump therapy had total daily insulin dose available from pump download for all time periods.

### Statistical analysis

Descriptive statistics were calculated for baseline (pre-ETI) demographic and clinical characteristics. Due skewness of absolute changes because of small sample size, medians, interquartile range (IQR) were reported for continuous variables, and frequencies and percentages were reported for categorical variables. CGM data were analyzed using the statistical computing software R (version 3.6.3) and R package CGM analysis (version 2.7.2) [20]. The CGM data were summarized during three (3-months) time periods: pre-ETI, 3-months post-ETI, and 6-months post- ETI. We evaluated changes from baseline using the Wilcoxon signed-rank test. For subgroup analyses, we used the Wilcoxon rank sum test to compare those changes by gender and by switching to ETI status. Statistical analyses were conducted using SAS (version 9.4). A significance level of p < 0.05 was used for all tests.

## Results

### Patients

Twelve patients with CFRD met inclusion criteria and were included in the study. All patients were on insulin therapy during the study window. Nine patients were on insulin pump therapy and 2 were on multiple daily injections (MDI). One patient was excluded due to stopping insulin after running out of medication during the 3-month period after starting ETI. The median age was 33 years (IQR 31–36 years). Of the 11 included in the study, 3 were males (27.3 %) and 8 were females (72.7 %). Two patients were on lumacaftor-ivacaftor prior to initiating ETI. Four patients were on tezacaftor-ivacaftor prior to initiating ETI. All patients were initiated on a standard dose of ETI. There was a statistically significant improvement in median FEV1 % predicted (P < 0.01), from pre-treatment 66 % (IQR 45–92 %) to 3 months post-treatment 60 % (IQR 40–80 %). There was a plateaued improvement at 6 months post-therapy, with a median 67 % (IQR 63–95 %; P = 0.06 compared to baseline). Median BMI was significantly higher at both 3- and 6-months post therapy (23.8 kg/m2, IQR: 21.9–25.7 kg/m2; 23.2 kg/m2, IQR: 22.3–26.3 kg/m2) compared to pretreatment (23.1 kg/m2, IQR: 21.6–24.8 kg/m2; P=<0.01 and P = 0.03, respectively). After 6-months of therapy, in patients with complete data sets there was a decline in median hemoglobin A1c (6.8 %, IQR:6.8–7.3 % vs baseline 7.1 %, IQR: 6.8–7.2), and a decline in median basal insulin requirement (6.8 units, IQR: 6.6–7.3 vs baseline 7.2 units, IQR: 6.6–8.2). However, those declines did not reach statistical significance (P = 0.88 and P = 0.15, respectively). Clinical characteristics of patients at baseline, 3-months post treatment and 6-months post treatment can be seen in Table 1.(see Table 2.).

**Table 1:** Clinical characteristics.

	Pre-ETI	3 Months post-ETI	6 Months post-ETI	Change from pre- to 3 Months post-ETI P^a^	Change from pre- to 6 Months post-ETI P^a^
Hemoglobin A1c (%) n = 5	7.1 (6.8–7.2)	7.2 (6.5–7.2)	6.8 (6.6–7.3)	>0.99	0.88
Basal (units)* n = 8	7.2 (6.6–8.2)	7.2 (6.5–7.4)	6.8 (6.6–7.3)	0.38	0.15
TDD (units) (pump patients) n = 1	12.6	10.9	10.0	NA	NA
FEV1 (% predicted) n = 11	60 (40–80)	66 (45–92)	67**** (63–95)	<0.01	0.06
BMI (kg/m^2^) n = 11	23.1 (21.6–24.8)	23.8 (21.9–25.7)	23.2 (22.3–26.3)	<0.01	0.03

Data reported as median (IQR).

ETI = elexacaftor-tezacaftor-ivacaftor; *Basal = includes pump and MDI; TDD = total daily dose.

FEV1 = Forced effective volume; BMI = Body mass index.

a Wilcoxon signed-rank test was used to test change in medians.

**** N = 5.

**Table 2:** Changes in CGM glycemic pattern pre- and post-Trikafta® initiation.

N = 11	Pre-Trikafta®	3 Months post- Trikafta®	6 Months post-Trikafta®	Change from pre- to 3 months post-ETI P	Change from pre-to 6 months post-ETI P
% Time CGM worn during sampling period	79 (60–91)	85 (74–95)	86 (77–94)	0.29	0.63
Length of CGM sampling period (days)	70 (51–86)	80 (57–88)	80 (49–87)	0.43	0.62
Sensor glucose (mg/dl)	153.6 (142.8–179.0)	147.4 (133.7–167.4)	143.0 (132.9–169.1)	0.24	0.52
% Time between 70 and 180 mg/dL	76.1 (56.4–77.6)	75.0 (64.4–87.6)	73.0 (63.0–87.8)	0.12	0.32
% Time < 54 mg/dL	0.19 (0.03–1.05)	0.14 (0.05–0.28)	0.13 (0.06–0.23)	0.32	0.83
% Time < 70 mg/dL	0.5 (0.2–4.7)	0.9 (0.5–1.8)	0.8 (0.5–1.7)	0.41	0.76
% Time > 140 mg/dL	51.3 (43.4–65.2)	46.1 (34.5–61.0)	43.2 (34.3–58.2)	0.10	0.15
% Time > 180 mg/dL	22.0 (20.2–44.3)	25.2 (11.7–35.3)	24.5 (11.5–37.2)	0.21	0.52
% time > 200 mg/dL	14.9 (12.9–29.9)	17.3 (6.1–27.5)	16.1 (6.6–27.6)	0.37	0.64
% time > 250 mg/dL	4.6 (3.2–10.1)	5.6 (1.1–9.8)	5.0 (1.3–10.3)	0.47	0.83
Number of excursions > 180 mg/dL	141 (118–192)	144 (98–193)	128 (74–181)	>0.99	0.62
Number of excursions > 250 mg/dL	34 (24–67)	46 (14–65)	36 (16–53)	0.76	0.48
Mean amplitude of glycemic excursions, calculated value (MAGE)	105.9 (98.6–109.2)	105.1 (81.9–124.8)	103.3 (82.0–124.8)	0.32	0.37

Data reported as median (IQR)

### CGM data

CGM data were collected for each patient over 3-months before (baseline) and two consecutive 3-month periods after starting ETI. In the 3-months period before initiation of ETI, the median sampling period was 70 days (IQR: 51–86 days), median percent time CGM was worn was 79 % (IQR 60–91 %). No significant differences between baseline and post treatment at 3- and 6-months were observed in sampling period (P = 0.43 and P = 0.62) or percent time CGM was worn (P = 0.29, and P = 0.63). Median sensor glucose was not significantly different at 3- or 6-months compared to baseline. Median glucose was 153.6 mg/dL (IQR 142.8–179.0 mg/dL) for the pre-treatment period compared to 147.4 mg/dL (IQR 133.7–167.4 mg/dL) at 3- months (P = 0.24) and 142 mg/dL (IQR 132.9–169.1 mg/dL) at 6- months (P = 0.52).

There was no significant difference in percent time spent in hyperglycemia or hypoglycemia between 3- months pretreatment and 3- or 6-months post treatment. When comparing baseline period to 3- and 6- months after initiation of therapy, percent time spent with a glucose > 180 mg/dL was 22.03 % (IQR: 20.2–44.3 %) vs 25.2 % (IQR: 11.7–35.3 %) at 3-months (P = 0.21), and 24.5 % (IQR: 11.5–37.2 %) at 6 months (P = 0.52); and percent time > 250 mg/dL was 4.6 % (IQR: 3.2–10.1 %) compared to 5.6 % (IQR: 1.1–9.8 %) at 3-months (P = 0.47), and 4.9 % (IQR: 1.3–10.3 %) at 6-months (P = 0.83). Less glucose excursions over 180 or 250 mg/dL were observed at 6-months post treatment compared to pretreatment, however, they did not reach statistical significance (P = 0.62 and P = 0.48, respectively).

We compared CGM data by gender and no statistically significant difference was seen in study subjects. When compared by previous treatment with modulator therapy, those who were new to start ETI had time lest spent with a glucose>120 mg/dL (P = 0.02) and>140 mg/dL at 3-months (P = 0.02 and P = 0.04, respectively).

## Discussion

In this retrospective study, we evaluated the effect of ETI on glycemia captured pre and post initiation of ETI in adults with CFRD using CGM. No significant changes in measures of glycemia were detected. After 6-months of therapy, there was a decline in median hemoglobin A1c and basal insulin requirements, however, those declines did not reach statistical significance. When analyzed by sex, there was no significant difference in glycemia between gender in our study. However, males had a trend towards improved glycemia as compared to females. This is consistent with prior study suggesting females with CFRD have poorer outcomes compared to their male counterparts. [32].

When exploring the subgroup of patients who were switching to from a different CFTR modulator, there was a trend towards improved glycemic control in those who were not previously on CFTR modulator therapy with a statistically significant improvement at 3-months in the percent of time spent with glucose values>120 mg/dL and 140 mg/dL.

Among patients with CF, studies show mixed results regarding effect of CFTR modulators on glycemia. In a study involving 40 patients, aged 12 years and older, with newly diagnosed diabetes or impaired glucose tolerance, Misgault and colleagues reported a decrease in CFRD by 50 % after one year of treatment with lumacaftor-ivacaftor. [21] Dagan et al., also reported similar results. [22] In a recent retrospective analysis following 14 adults with CFRD on ivacaftor therapy alone or in combination over 2.5 years, 5 patients were able to discontinue insulin therapy. [23] Moreover, case reports suggest an improvement in glycemia and remission of CFRD with sustained glycemic control off insulin after treatment with ivacaftor. [10], [12] In addition, large observational data from the US and UK CF registry demonstrated a lower prevalence of CFRD among CF patients treated with CFTR modulator therapy compared with an untreated control with similar genotype severity. [13] However, there is also evidence that CFTR modular therapy has no effect on glycemia. Moheet and colleagues analyzed a subgroup of 39 subjects from the PROSPECT study, a clinical trial investigating the effect of lumacaftor/ivacaftor on lung function, found no improvement in insulin secretion or glucose tolerance testing after one year of therapy among subjects with normal and abnormal glucose tolerance as well as those with CFRD. [24], [25] Thomassen et al., reported similar results after 8 weeks of treatment in patients homozygous for Phe508del mutation. [26].

CGM is validated and has been studied among CF adults and youth with and without diabetes. [16] The % time in range of 70–180 mg/dL is the recommended treatment goal for patients with type 1 and type 2 diabetes as well as people with CF. [32] The CGM data captured in our study was practical in that it reflects real-life data points. The median CGM wear was 70 days pre- ETI and 80 days during the 3-month and 6-month post- ETI periods. This allowed us to capture a more comprehensive view of glycemic trends over time rather than a snapshot.

Our results are in concordance with those shown by Wood et al. where they retrospectively examined real life CGM data in 12 patients, mostly pediatric, with CFRD for about 9 months before and 7 months after initiation of ETI and similar to our study did not show significant improvement in CGM data or change in hypoglycemia. [19] In CF youth without diabetes, Li and colleagues utilized CGM over a median of 29 months of therapy with lumacaftor-ivacaftor found no difference in serum glucose or insulin secretion. Nevertheless, male patients showed lower glycemic variability after treatment (P = 0.03). [17] Scully and colleagues prospectively studied 14 adults with CFRD using Free Style Libre CGM® for 14 days prior to initiation of ETI and 14 days within one year after (patients were blinded to CGM data). [18] Their results showed an improvement in % time between glucose 70–180 mg/dL and % time spent glucose>200 mg/dL in patients with CFRD within 12 months of initiating ETI.

Hypoglycemia has been reported as a side effect of CFTR modulators. [23] However, we detected no difference in hypoglycemic events, < 70 mg/dL or < 54 mg/dL similar to what is seen in other studies. [18], [19] It is possible that hypoglycemic events occurred shortly after initiating ETI and may have been missed as no pre-defined window for capturing data points was set for the immediate period after initiating therapy.

Our study has several limitations that may have influenced the results. Although more representative of the real world, the extended CGM sampling period may have led to greater variability which affected measures of glycemia. Our CGM data were not blinded to the patient, which may have also played a role. For many patients, ETI. was initiated in the late Fall 2019, and the 3-month and 6-month follow up period after occurred during the early part of 2020 during the height of the COVID-19 pandemic. Some patients did not follow up during this time. Therefore, some parameters – A1c, basal units of insulin, total daily insulin dose were not taken. Some clinic visits during this time were done virtually, and some measures of weight and spirometry were performed with home measures. ETI therapy was managed by patients’ pulmonary team and assessment of compliance was limited to chart review.

Finally, diet intake was not tracked during this study period. It is unclear if increased caloric intake during the study period influenced CGM data especially during COVID-19 pandemic.

Our study is strong due to representing real world CGM data using patients’ personal CGM devices sampled for long duration, however, was limited by small sample size and retrospective design.

## Conclusion

The impacts of ETI on glycemia were examined as measured by CGM in 12 adults with CFRD at our clinic without improvement in glycemic control or change in hypoglycemia. Future, prospective studies on CFTR modulators are needed to determine whether this class of medication impacts β-cell function and glucose metabolism in patients with and without CFRD.

## Declaration of Competing Interest

The authors declare that they have no known competing financial interests or personal relationships that could have appeared to influence the work reported in this paper.
